# Patterns of *de novo* metastasis and survival outcomes by age in breast cancer patients: a SEER population-based study

**DOI:** 10.3389/fendo.2023.1184895

**Published:** 2023-11-06

**Authors:** Qian Xiao, Weixiao Zhang, Jingfeng Jing, Tingting Zhong, Daxue Li, Jing Zhou, Pan Liu, Zhongxu Duan, Han Gao, Liyuan Shen

**Affiliations:** ^1^ Department of Breast and Thyroid Surgery, Women and Children’s Hospital of Chongqing Medical University, Chongqing, China; ^2^ Department of Breast and Thyroid Surgery, Chongqing Health Center for Women and Children, Chongqing, China; ^3^ Department of Nutrition, Chongqing Jiangbei Hospital of traditional Chinese medicine, Chongqing, China; ^4^ Department of Cardiology, Chongqing General Hospital, Chongqing, China; ^5^ Department of Rheumatology, Daping Hospital, the Third Affiliated Hospital of Third Military Medical University, Chongqing, China; ^6^ Department of Obstetrics and Gynecology, Women and Children’s Hospital of Chongqing Medical University, Chongqing, China; ^7^ Department of Obstetrics and Gynecology, Chongqing Health Center for Women and Children, Chongqing, China

**Keywords:** *de novo* metastatic breast cancer, age, overall survival, breast cancer-specific survival, SEER

## Abstract

**Background:**

The role of age in metastatic disease, including breast cancer, remains obscure. This study was conducted to determine the role of age in patients with *de novo* metastatic breast cancer.

**Methods:**

Breast cancer patients diagnosed with distant metastases between 2010 and 2019 were retrieved from the Surveillance, Epidemiology, and End Results database. Comparisons were performed between young (aged ≤ 40 years), middle-aged (41–60 years), older (61–80 years), and the oldest old (> 80 years) patients. Adjusted hazard ratios (aHRs) and 95% confidence intervals (CIs) were estimated using multivariate Cox proportional hazard models. Survival analysis was performed by the Kaplan–Meier method.

**Results:**

This study included 24155 (4.4% of all patients) *de novo* metastatic breast cancer patients. The number of young, middle-aged, older, and the oldest old patients were 195 (8.3%), 9397 (38.9%), 10224 (42.3%), and 2539 (10.5%), respectively. The 5-year OS rate was highest in the young (42.1%), followed by middle-aged (34.8%), older (28.3%), and the oldest old patients (11.8%). Multivariable Cox regression analysis showed that middle-aged (aHR, 1.18; 95% CI, 1.10–1.27), older (aHR, 1.42; 95% CI, 1.32–1.52), and the oldest old patients (aHR, 2.15; 95% CI, 1.98–2.33) had worse OS than young patients. Consistently, middle-aged (aHR, 1.16; 95% CI, 1.08–1.25), older (aHR, 1.32; 95% CI, 1.23–1.43), and the oldest old patients (aHR, 1.86; 95% CI, 1.71–2.03) had worse BCSS than young patients.

**Conclusion:**

This study provided clear evidence that *de novo* metastatic breast cancer had an age-specific pattern. Age was an independent risk factor for mortality in patients with *de novo* metastatic breast cancer.

## Introduction

Breast cancer is the most frequently diagnosed malignancy in women, with an estimated 2.3 million new cases (11.7%) in 2020 worldwide (1). After lung cancer, breast cancer is the second leading cause of cancer-associated deaths in women (2). Among the new breast cancer cases, 2.4%–6% of patients were diagnosed with advanced breast cancer with stage IV, namely, *de novo* metastatic breast cancer (3, 4). According to the data from 2009 to 2015 in the United States, patients with *de novo* metastatic breast cancer had the lowest 5-year overall survival (OS) rate (27%), which was much lower than stage I (98%), stage II (92%), and stage III (75%) (5). Identifying clinical risk factors that are closely correlated with distant metastases may help provide clues for the underlying mechanism of distant metastases and the development of treatment strategies for advanced breast cancer.

Cancer cells disseminated from the primary tumor before detection and persist at distant organs are thought to be the source of distant metastasis (6). The classic model of metastasis includes three steps: first, the primary tumor grows and infiltrates vessels; second, cancer cells enter the blood or lymphatic vessels and spread to distant sites; and third, disseminated cells colonize new sites and outgrowths of metastases (7). Only a small percentage of disseminated tumor cells emerging from step one acquire sufficient genetic or epigenetic alterations that enable them to complete the metastasis (8). The latency between tumor cell dissemination and outgrowth of metastases varies from a very short period (early metastasis) to decades, which experience a long-term democracy (9). According to epidemiologic studies, some sociodemographic and clinicopathologic features are found to be risk factors associated with metastases. For example, it is well-known that patients with triple-negative breast cancers (TNBC) have an early tendency to undergo metastasis and a higher tumor recurrence rate than other types of breast cancers (10, 11). Further studies focusing on TNBC revealed mechanisms and targets for distant metastasis that contributed to an improvement in survival outcomes among patients with TNBC (10).

Age, as a major risk factor of tumorigenesis, contributes greatly to metastasis, likely because of the age-related changes in patient homeostasis and the tumor microenvironment (12). Distant metastases occur earlier and are more synchronized in older patients than in younger patients with melanoma (13). Age-induced reprogramming of lung fibroblasts enables metastatic outgrowth of melanoma in the lungs by increasing the secretion of secreted frizzled-related protein 1 (sFRP1) (14). In breast cancer, age has been identified as a major risk factor for tumor initiation, metastasis, and disease-specific mortality (15). Young breast cancer patients usually exhibit more aggressive tumor characteristics, while older patients often have a worse prognosis (16, 17); however, whether there is a close relationship between age and metastasis in breast cancer patients remains largely elusive. In this retrospective study, we systemically evaluated the association among different age groups with *de novo* metastatic breast cancer. The clinicopathologic characteristics and the risk of all-cause mortality and breast cancer-specific mortality were also determined in *de novo* metastases based on patient age at the time of diagnosis.

## Methods

### Data source

The cohort of this study was collected from the most recent Surveillance, Epidemiology, and End Results (SEER) 17 registries’ (November 2021) database (https://seer.cancer.gov/) using SEER*Stat 8.4.0.1. The SEER is a population-based dataset that covers approximately 30% of the United States, providing demographic, clinicopathologic, diagnostic, treatment, and survival information. Due to the publicity of the SEER database, the current study was exempt from the review of the Institutional Ethics Committees of the Women and Children’s Hospital of Chongqing Medical University. Because no participants were enrolled in this study, written informed consent from patients was waived. This study followed the Strengthening the Reporting of Observational Studies in Epidemiology (STROBE) reporting guideline.

### Study population

**Figure 1:**
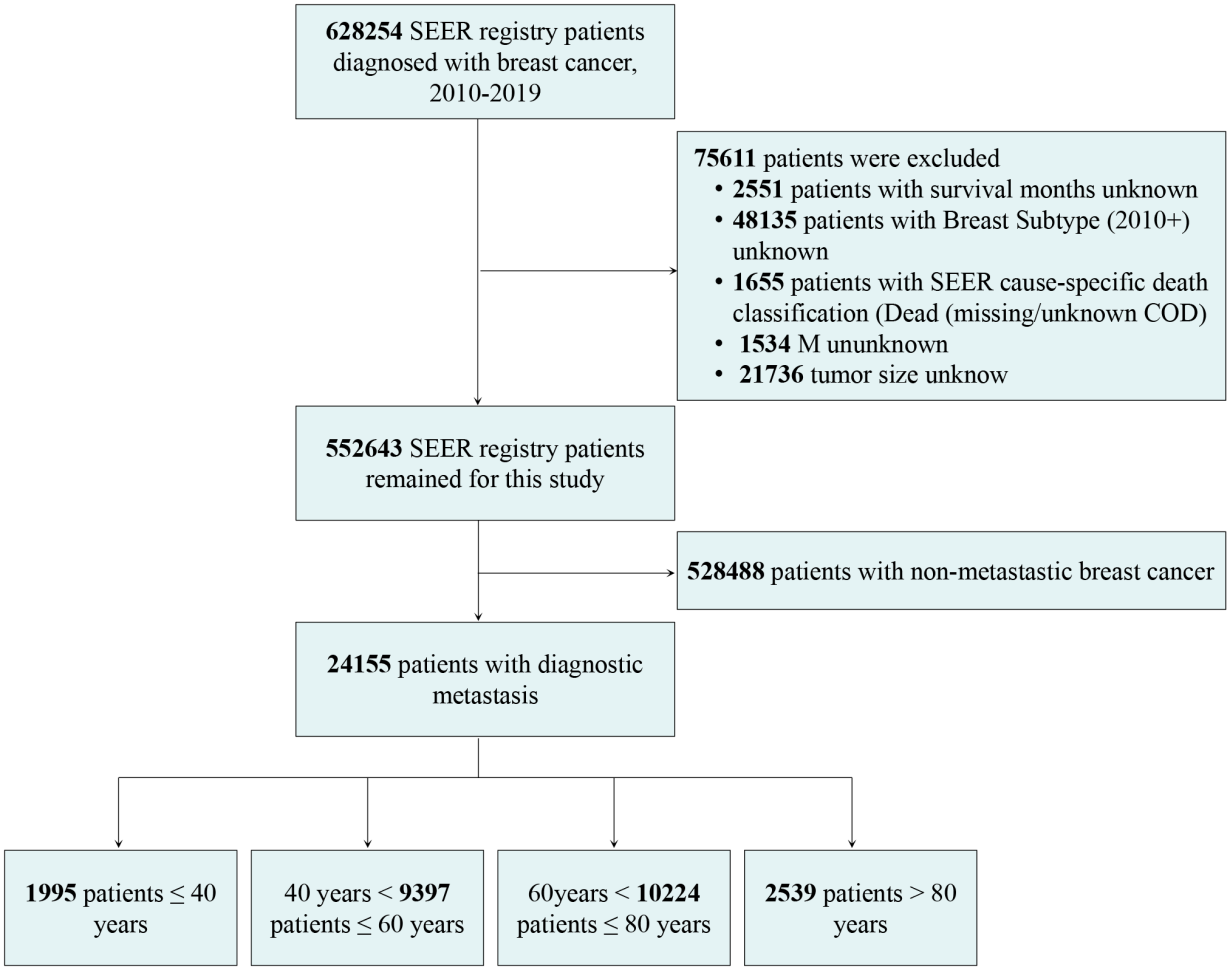
Flow chart of patient selection.

Patients diagnosed with *de novo* metastatic breast cancer between 2010 and 2019 were included because the human epidermal growth factor receptor 2 (HER2) status and breast cancer subtype data were available since 2010. A total of 628254 breast cancer patients were originally identified without other restrictions. We obtained demographic variables, clinicopathologic features, and treatment information from the SEER, including patient age at the time of diagnosis, sex, year of diagnosis, race, tumor size, lymph node status, distant metastases, tumor grade, histologic type, molecular subtype, surgery, radiotherapy, chemotherapy, survival in months, and cause of death. Patients were excluded if the following critical information was unknown: survival months, breast subtype (2010+), cause-specific death classification, metastases, and tumor size. The flow chart for patient selection is shown in **Figure 1**.

Individuals were grouped into young (aged ≤ 40 years), middle-aged (41–60 years), older (61–80 years), and the oldest old (> 80 years) based on the patient’s age at the time of diagnosis. Tumor size was categorized as <2 cm, 2–5 cm, >5 cm, or unknown. According to the 3rd edition of the International Classification of Diseases for Oncology (ICD-O-3), histopathology codes and histology types were grouped into invasive ductal carcinoma (IDC, 8500/3), invasive lobular carcinoma (ILC, 8520/3), mixed IDC and ILC (8522/3), or other types. We stratified surgery into “yes,” “no,” and “unknown” groups according to the 2021 breast surgery codes from SEER. Specifically, the chemotherapy recode no/unknown represented patients who did not receive chemotherapy because the recode indicated that no evidence of chemotherapy was identified in the medical records. Radiotherapy was categorized into either a "yes" or "no/unknown" group based on the radiation code. Patients who either did not require radiotherapy, refused it, or had an unknown treatment status were consolidated into the "no/unknown" group. Information on hormonal or target therapy was not provided. Our primary outcome was OS based on the vital status recode variation, which was defined as any cause of death. In addition, breast cancer-specific survival (BCSS), as a secondary outcome, was also determined using the SEER cause-specific death classification. BCSS was considered a death caused by breast cancer. The variable survival time months were used to extract information on the follow-up period.

### Statistical analysis

Demographic and clinicopathologic characteristics of patients were examined by descriptive statistics and/or chi-square test. Kaplan-Meier survival curves were carried out to assess OS and BCSS. The differences between survival curves were detected by log-rank tests. We performed a univariable (unadjusted) analysis of the hazard ratio (HR) and 95% confidence intervals (CIs) of OS and/or BCSS using the Mantel-Haenszel statistic. Multivariable Cox proportional hazards regression models were used to assess the influence of patient age at the time of diagnosis on the HRs of OS and BCSS after adjusting for sex, race, tumor size, lymph node status, distant metastases, tumor grade, molecular subtype, histologic type, surgery, chemotherapy, and radiotherapy. The proportional hazards model assumption was confirmed for each covariate by inspecting log (-log [survival]) curves. All *P*-values were two-sided with a statistical significance of *P* < 0.05. All statistical analyses were performed using GraphPad Prism version 8.0.1 (GraphPad Software Inc, La Jolla, CA, USA) and IBM SPSS 22.0 software (IBM Corporation, Armonk, NY, USA).

## Results

### Baseline characteristics

A total of 24155 (4.4% of all breast cancer patients) *de novo* metastatic breast cancer patients (mean [SD] age, 61.5 [14.4] years [range, 15-100 years]) were included in this study (**Figure 1**). The median follow-up was 20 months (range, 0–119) for all patients. There were 12564 (90%) death events attributed to breast cancer and 1394 (10%) deaths due to other causes. The number of patients ≤ 40 (young), 41–60 (middle-aged), 61–80 (older), and >80 years (the oldest old) were 1995 (8.3%), 9397 (38.9%), 10224 (42.3%), and 2539 (10.5%), respectively. There were 15740 (65.2%) patients with bone, 7148 (29.6%) lung, 5796 (24.0%) liver, and 1599 (6.6%) brain metastases. A total of 7550 patients underwent surgery for primary tumors after diagnosis with metastatic breast cancer. The demographic and clinical characteristics of the patients are shown in **Table 1**. Among the patients with *de novo* metastatic breast cancer, young white patients had the lowest percentage (66.8%), followed by middle-aged (70.5%), older (78.5%), and the oldest old (85.2%), whereas young Black patients had the highest percentage (21.3%), followed by middle-aged (17.8%), older (14.0%), and the oldest old (8.7%). The oldest old patients with *de novo* metastatic breast cancer were most frequently associated with lymph node negativity (33%), while lymph node positivity was more frequent in young patients (70.8%). With respect to distant metastases, young patients had the highest proportion of bone (66.3%) and liver (33.6%) metastases. The proportions of patients with lung metastases gradually increased from young (21.8%) to middle (27.1%) to older (32.4%) to the oldest old (33.6%) patients. The percentage of patients with brain metastasis had higher percentages in the middle (7.2%) and older patients (7.0%) than the young (5.7%) and oldest old patients (3.5%). Surgery, chemotherapy, and radiotherapy were administered more often to young patients, followed by middle-aged, older, and the oldest old patients (**Table 1**).

**Table 1:** Characteristics of patients stratified by age, SEER, 2010–2019.

Characteristics	All patients		≤40		41-60		61-80		>80		*P*-value^a^
	N	%	N	%	N	%	N	%	N	%	
All patients	24155	100.0	1995	8.3	9397	38.9	10224	42.3	2539	10.5	
Sex											
Male	297	1.2	9	0.5	104	1.1	153	1.5	31	1.2	<0.001
Female	23858	98.8	1986	99.5	9293	98.9	10071	98.5	2508	98.8	
Year of diagnosis											
2010-2014	11121	46.0	879	44.1	4602	49.0	4562	44.6	1078	42.5	<0.001
2015-2019	13034	54.0	1116	55.9	4795	51.0	5662	55.4	1461	57.5	
Race											
White	18140	75.1	1332	66.8	6623	70.5	8023	78.5	2162	85.2	<0.001
Black	3747	15.5	425	21.3	1674	17.8	1427	14.0	221	8.7	
Other	2173	9.0	228	11.4	1055	11.2	739	7.2	151	5.9	
Unknown	95	0.4	10	0.5	45	0.5	35	0.3	5	0.2	
Tumor size											
≤2.0	4770	19.7	309	15.5	1798	19.1	2168	21.2	495	19.5	<0.001
2.0-5.0	10990	45.5	930	46.6	4225	45.0	4515	44.2	1320	52.0	
>5	8395	34.8	756	37.9	3374	35.9	3541	34.6	724	28.5	
Lymph node											
N0	5694	23.6	325	16.3	1884	20.0	2646	25.9	839	33.0	<0.001
N1	10842	44.9	980	49.1	4414	47.0	4442	43.4	1006	39.6	
N2	2620	10.8	243	12.2	1093	11.6	1059	10.4	225	8.9	
N3	3637	15.1	377	18.9	1583	16.8	1436	14.0	241	9.5	
NX	1362	5.6	70	3.5	423	4.5	641	6.3	228	9.0	
Bone metastasis											
Yes	15740	65.2	1323	66.3	6192	65.9	6721	65.7	1504	59.2	<0.001
No	8117	33.6	655	32.8	3108	33.1	3367	32.9	987	38.9	
Unknown	298	1.2	17	0.9	97	1.0	136	1.3	48	1.9	
Lung metastasis											
Yes	7148	29.6	435	21.8	2548	27.1	3311	32.4	854	33.6	<0.001
No	16437	68.0	1528	76.6	6636	70.6	6676	65.3	1597	62.9	
Unknown	570	2.4	32	1.6	213	2.3	237	2.3	88	3.5	
Liver metastasis											
Yes	5796	24.0	671	33.6	2588	27.5	2090	20.4	447	17.6	<0.001
No	17911	74.2	1299	65.1	6660	70.9	7929	77.6	2023	79.7	
Unknown	448	1.9	25	1.3	149	1.6	205	2.0	69	2.7	
Brain metastasis											
Yes	1599	6.6	114	5.7	677	7.2	718	7.0	90	3.5	<0.001
No	21994	91.1	1846	92.5	8519	90.7	9257	90.5	2372	93.4	
Unknown	562	2.3	35	1.8	201	2.1	249	2.4	77	3.0	
Grade											
I	1435	5.9	57	2.9	485	5.2	707	6.9	186	7.3	<0.001
II	7208	29.8	497	24.9	2769	29.5	3134	30.7	808	31.8	
III	8082	33.5	847	42.5	3469	36.9	3031	29.6	735	28.9	
IV	92	0.4	15	0.8	33	0.4	37	0.4	7	0.3	
Unknown	7338	30.4	579	29.0	2641	28.1	3315	32.4	803	31.6	
Histology											
IDC 8500/3	17705	73.3	1674	83.9	7101	75.6	7174	70.2	1756	69.2	<0.001
ILC 8520/3	2540	10.5	77	3.9	866	9.2	1288	12.6	309	12.2	
Mixed IDC/ILC 8522/3	1084	4.5	79	4.0	430	4.6	465	4.5	110	4.3	
Other types	2826	11.7	165	8.3	1000	10.6	1297	12.7	364	14.3	
Molecular subtype											
HR+/HER2-	14956	16.2	972	48.7	5461	58.1	6763	66.1	1760	69.3	<0.001
HR+/HER2+	3903	61.9	481	24.1	1702	18.1	1433	14.0	287	11.3	
HR-/HER2+	1980	8.2	229	11.5	912	9.7	710	6.9	129	5.1	
HR-/HER2-	3316	13.7	313	15.7	1322	14.1	1318	12.9	363	14.3	
Surgery											
Yes	7550	31.3	838	42.0	3252	34.6	2892	28.3	568	22.4	<0.001
No	16091	66.6	1100	55.1	5909	62.9	7128	69.7	1954	77.0	
Unknown	514	2.1	57	2.9	236	2.5	204	2.0	17	0.7	
Chemotherapy											
Yes	14746	61.0	1687	84.6	6784	72.2	5710	55.8	565	22.3	<0.001
No	9409	39.0	308	15.4	2613	27.8	4514	44.2	1974	77.7	
Radiotherapy											
Yes	7657	31.7	793	39.7	3361	35.8	3010	29.4	493	19.4	<0.001
No/Unknown	16498	68.3	1202	60.3	6036	64.2	7214	70.6	2046	80.6	
Vital status											
Alive	10197	42.2	1083	54.3	4327	46.0	4147	40.6	640	25.2	<0.001
Dead of breast cancer	12564	52.0	873	43.8	4757	50.6	5384	52.7	1550	61.0	
Dead of other cause	1394	5.8	39	2.0	313	3.3	693	6.8	349	13.7	

IDC, invasive ductal carcinoma; ILC, invasive lobular carcinoma.

a Calculated using Pearson’s chi-square test.

**Figure 2:**
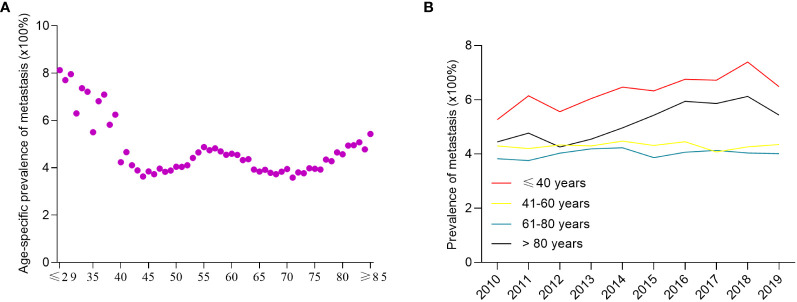
The prevalence of *de novo* metastatic breast cancer among all breast cancer patients. The age-specific prevalence of *de novo* metastatic breast cancer **(A)** and year-specific prevalence of *de novo* metastatic breast cancer **(B)**.

When analyzed by age, the prevalence of *de novo* metastatic breast cancer in overall breast cancer patients had a “w”-like shape (**Figure 2A**), which was highest in patients aged ≤29 years of age (8.1%), and then decreased to patients aged 44 years old (3.6%). There was a peak in 55-year-old patients (4.8%). The prevalence of *de novo* metastatic breast cancer gradually decreased from 4.8% (55 years of age) to 3.7% (68 years of age), and then increased up to 5.4% in patients ≥85 years of age. Between 2010 and 2019, the prevalence of *de novo* metastatic breast cancer patients ≤ 40 years of age increased from 5.3% to 6.5% and from 4.4% to 5.4% in patients >80 years of age, while the prevalence of *de novo* metastatic breast cancer was stable among patients 41–60 and 61-80 years of age with an average prevalence of 4.3% and 4.0%, respectively (**Figure 2B**).

**Figure 3:**
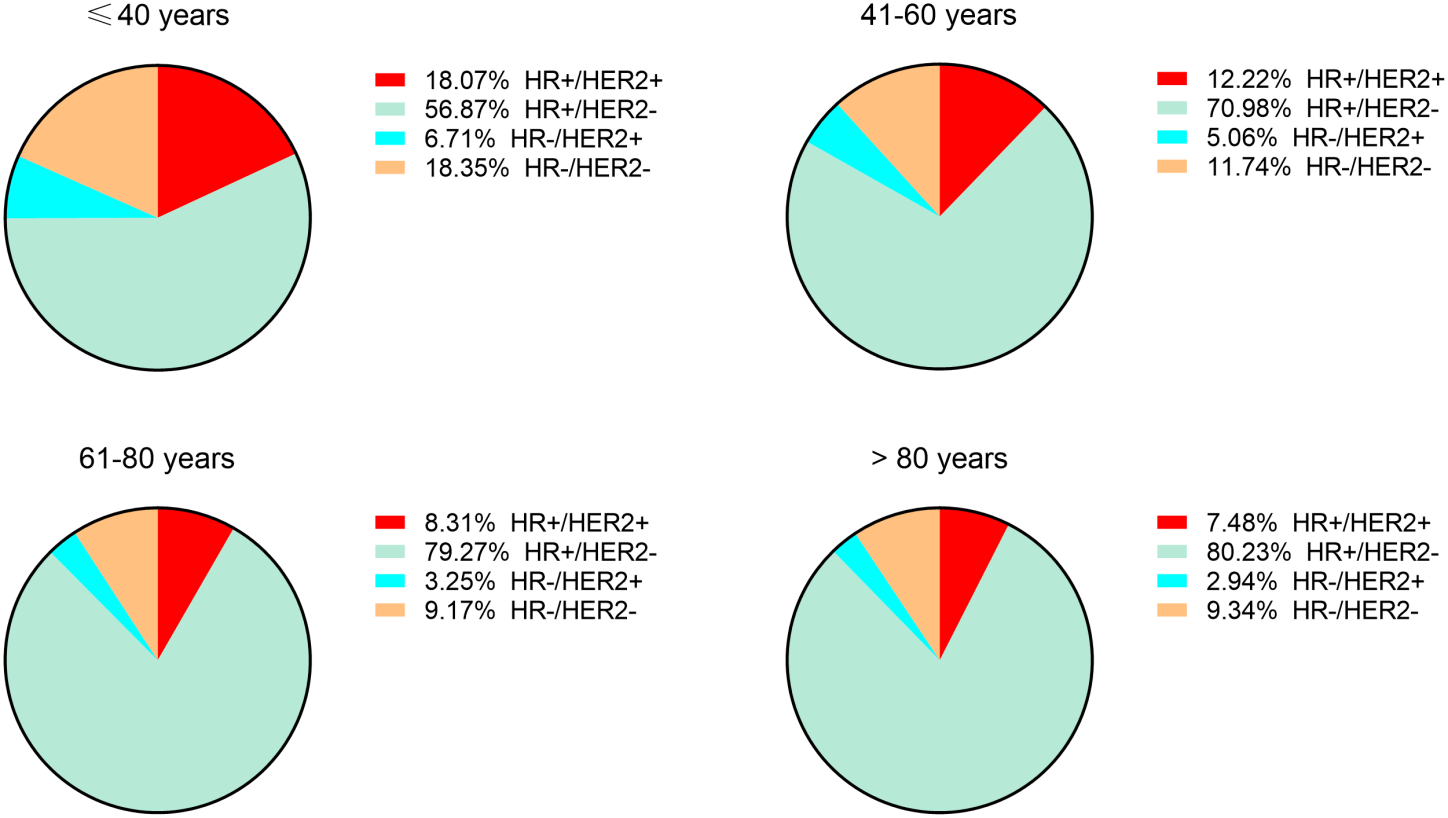
The percentages of four molecular subtypes among all breast cancer patients.

**Figure 4:**
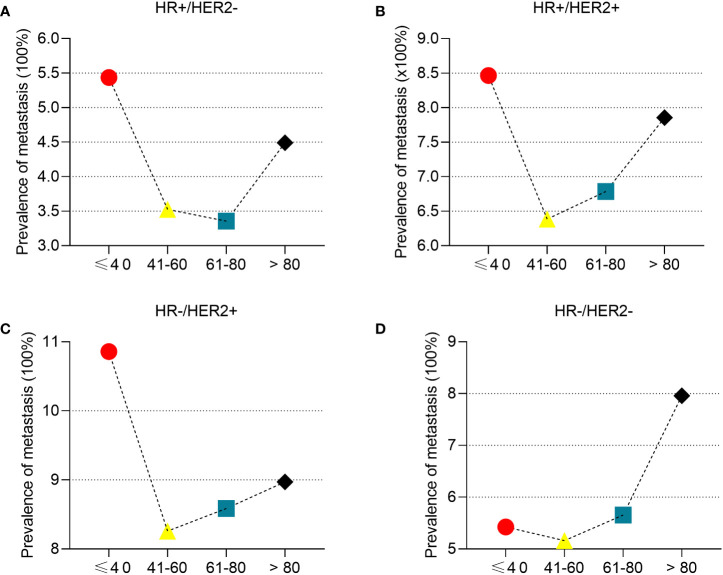
The prevalence of *de novo* metastasis in different ages stratified by subtypes of breast cancer. HR+/HER2- **(A)**, HR+/HER2+ **(B)**, HR-/HER2+ **(C)**, and HR-/HER2- **(D)**.

**Figure 5:**
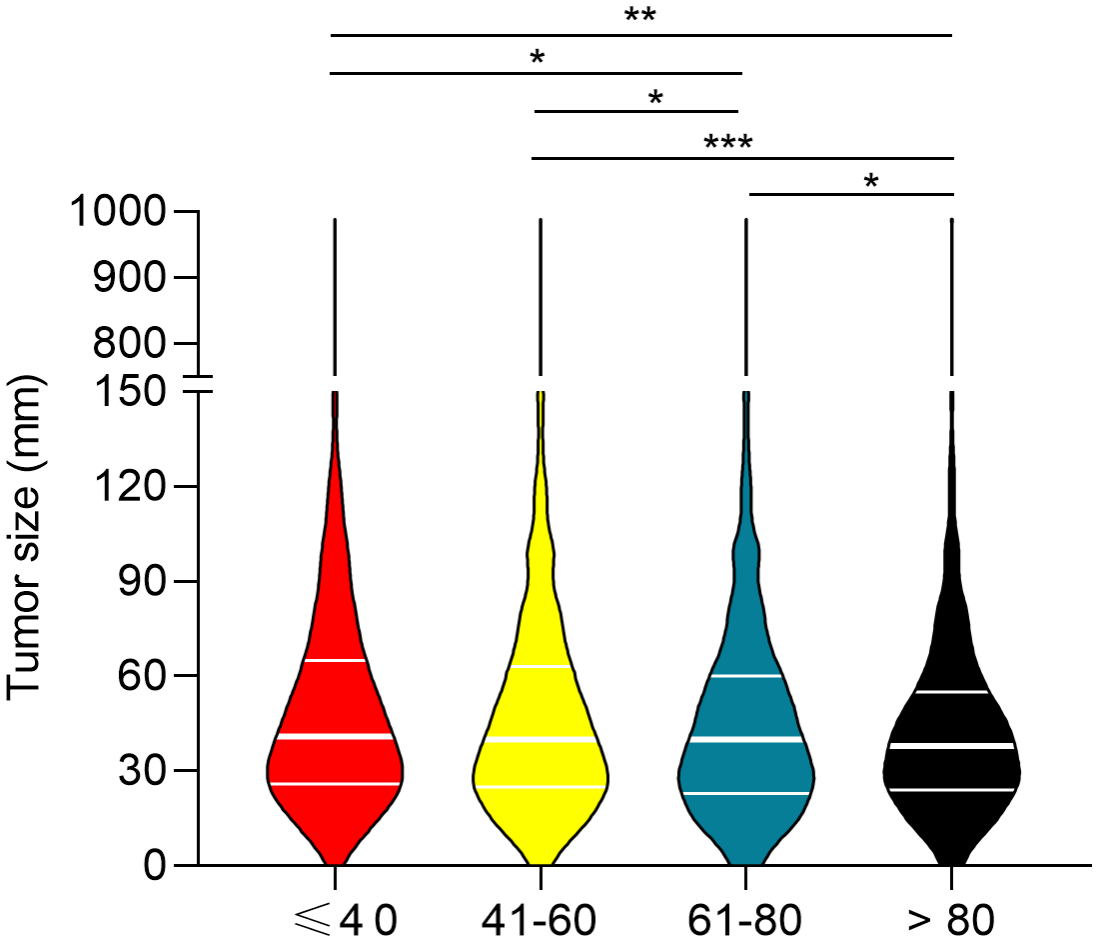
Comparison of primary tumor size between the four age groups. ^*^P<0.05, ^**^P<0.01, and ^***^P<0.001.

To determine the correlation between breast cancer subtype and metastasis in different age groups, we first analyzed the percentages of four molecular subtypes in the overall cohort, including early breast cancer patients (**Figure 3**). Patients ≤ 40 years of age had the highest percentage of HR+/HER2+ (18.07%), HR-/HER2+ (6.71%), and HR-/HER2- (18.35%) breast cancer molecular subtypes, whereas patients >80 years of age had the highest percentage of the HR+/HER2- (80.23%) breast cancer. Subsequently, we determined the prevalence of *de novo* metastases in different subtypes of breast cancer. Young patients had the highest prevalence of *de novo* metastasis in HR+/HER2- (**Figure 4A**), HR+/HER2+ (**Figure 4B**), and HR-/HER2+ (**Figure 4C**) molecular subtypes, followed by the oldest old, older, and/or middle-aged patients. The oldest old patients had the highest prevalence of HR-/HER2- breast cancer, followed by older, young, and middle-aged patients (**Figure 4D**). To determine the correlation between primary tumor burden and *de novo* metastasis, we compared the tumor sizes among the four age groups. The oldest old patients had the smallest primary tumor size (mean [SD] = 45.7 [62.1] mm), followed by the older (mean = 48.1 [53.0] mm), middle-age (mean = 49.8 [51.4] mm), and young patients (mean = 50.8 [44.9] mm) (*P* < 0.001, **Figure 5**).

### Survival analysis

**Figure 6:**
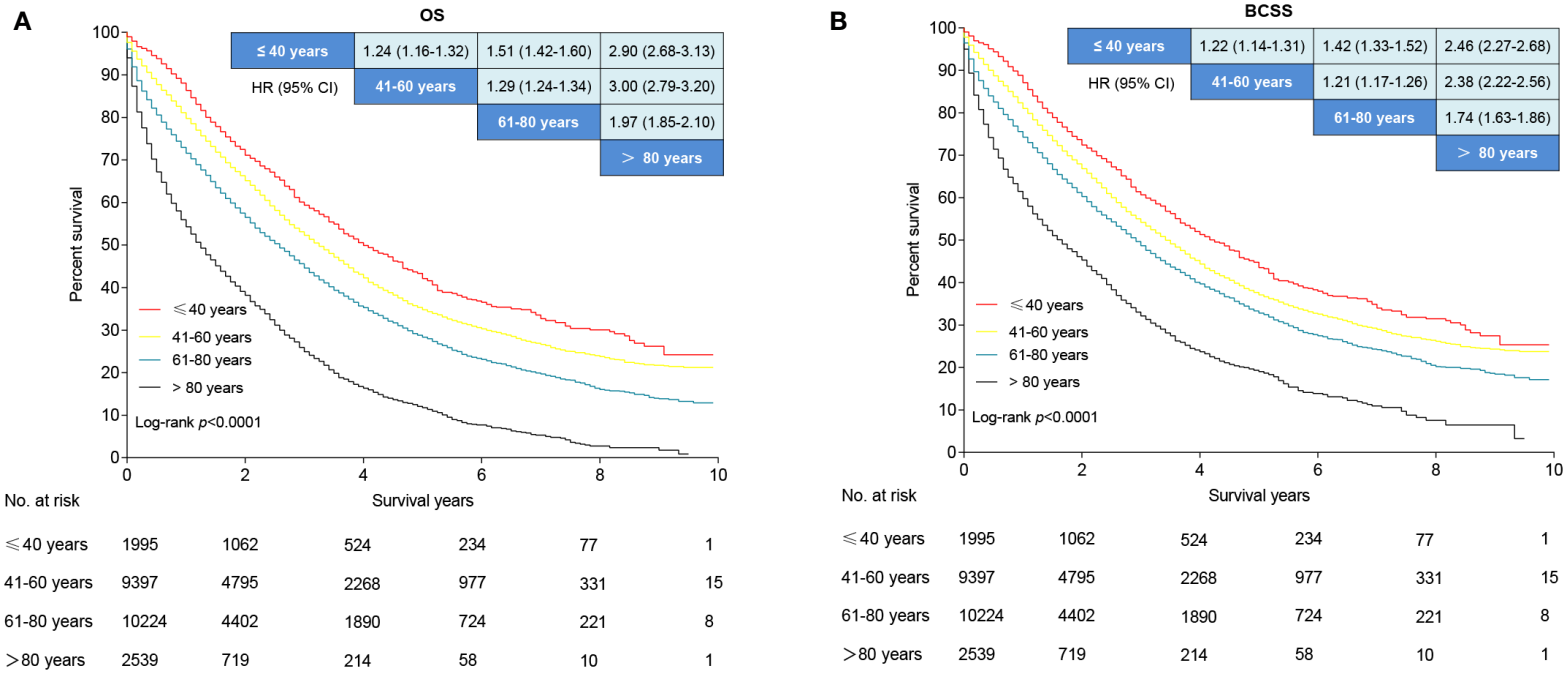
Survival analysis of *de novo* metastatic breast cancer stratified by age. **(A)** Overall survival curves. **(B)** Breast cancer-specific survival curves. Data are presented as hazard ratios and 95% confidence intervals in the league table of comparisons. An HR >1 favors the row-defining group.

The median follow-up period was 2.2 (range 0 to 9.9), 2.0 (range 0 to 9.9), 1.6 (range 0 to 9.9), and 0.9 (range 0 to 9.5) years for young-age, middle-age, older-age, and the oldest old patients, respectively. The 5-year OS rates were 42.1%, 34.8%, 28.3%, and 11.8% for young-age, middle-aged, older-age, and the oldest old patients, respectively. Patients in the oldest old group had the worst OS compared to the young-age group (HR, 2.90; 95% CI, 2.68–3.13; *P* < 0.001), middle-age (HR, 3.00; 95% CI, 2.97–3.20; *P* < 0.001), and older-age (HR, 1.97; 95% CI, 1.85–2.10; *P* < 0.001) based on unadjusted analysis (**Figure 6A**). Older-aged patients had a significantly poorer OS than middle-aged (HR, 1.29; 95% CI, 1.24–1.34; *P* < 0.001) and young-aged patients (HR, 2.17; 95% CI, 1.61–2.92; P <.001; **Figure 6A**). Middle-aged patients had a decreased OS compared to young-aged patients (HR, 1.24; 95% CI, 1.61–1.32; P < 0.001; **Figure 6A**). Older-aged patients had decreased OS compared to young-aged (HR, 1.51; 95% CI, 1.42–1.60; P < 0.001) and middle-aged patients (HR, 1.29; 95% CI, 1.24–1.34; P < 0.001; **Figure 6A**). Similar BCSS differences were noted among the four age groups of patients (**Figure 6B**).

Subgroup analyses were performed based on subtypes, metastasis sites, and treatment (**Supplementary Figures 1**–**6**). The OS and BCSS were best in the young patients with HR+/HER2-, HR-/HER2+, and HR-/HER2- molecular subtypes, followed by the middle-aged, older, and the oldest old patients (**Supplementary Figures 1A-C**, **2A-C**). There was no significant difference in OS and BCSS between the young- and middle-aged patients with TNBC; however, the oldest old patients had worse OS and BCSS than the other age groups (**Supplementary Figures 1D**, **2D**). In patients with bone and liver metastasis, young patients had the best OS and BCSS followed by middle-aged, older, and the oldest old patients (**Supplementary Figures 3A, B**, **4A, B**). No significant differences in OS and BCSS were noted between the young- and middle-aged patients with lung and brain metastases, whereas the two subgroups had significantly better OS and BCSS than older and the oldest old patients (**Supplementary Figures 3C, D**, **4C, D**). Among patients who received chemotherapy or surgery, the young patients had the best OS and BCSS, followed by the middle-aged, older, and oldest old patients (**Supplementary Figures 5**, **6**).

In multivariable analysis, middle-aged (HR, 1.18; 95% CI, 1.10–1.27; *P* < 0.001), older age (HR, 1.42; 95% CI, 1.32–1.52; *P* < 0.001), and the oldest old patients (HR, 2.15; 95% CI, 1.98-2.33; *P* < 0.001) had an 18%, 42%, and 115% higher risk of all-cause death rates compared with the young patients, respectively (**Table 2**). The oldest old patients (HR, 1.86; 95% CI, 1.71–2.03; *P* < 0.001) had the worst BCSS, followed by older age (HR, 1.32; 95% CI, 1.23–1.43; *P* < 0.001) and middle-aged patients (HR, 1.16; 95% CI, 1.08–1.25; *P* < 0.001) compared to the young patients (**Table 2**).

**Table 2:** Multivariable Cox regression for OS and BCSS.

Variable	OS			BCSS		
	Hazard Ratio	95% CI	*p*-value	Hazard Ratio	95% CI	*p*-value
Age at diagnosis						
≤40	1.00	Reference		1.00	Reference	
41-60	1.18	1.10-1.27	<0.001	1.16	1.08-1.25	<0.001
61-80	1.42	1.32-1.52	<0.001	1.32	1.23-1.43	<0.001
>80	2.15	1.98-2.33	<0.001	1.86	1.71-2.03	<0.001
Sex						
Female	1.00	Reference		1.00	Reference	
Male	1.18	1.02-1.37	0.03	1.19	1.01-1.39	0.35
Race						
White	1.00	Reference		1.00	Reference	
Black	1.37	1.35–1.39	<0.001	1.30	1.24-1.36	<0.001
Other	0.94	0.89-1.00	0.07	0.95	0.89-1.02	0.14
Unknown	0.39	0.25-0.61	<0.001	0.41	0.26-0.66	<0.001
Year of diagnosis	0.98	0.97-0.99	<0.001	0.98	0.97-0.98	<0.001
Tumor size						
T0	1.00	Reference		1.00	Reference	
T1	1.33	1.17-1.52	<0.001	1.40	1.21-1.61	<0.001
T2	1.49	1.31-1.69	<0.001	1.57	1.37-1.80	<0.001
T3	1.60	1.40-1.82	<0.001	1.70	1.48-1.96	<0.001
T4	1.82	1.60-2.06	<0.001	1.92	1.68-2.21	<0.001
Unknown	1.82	1.57-2.10	<0.001	1.91	1.64-2.24	<0.001
Node positivity						
N0	1.00	Reference		1.00	Reference	
N1	0.98	0.93-1.02	0.27	0.99	0.94-1.04	0.64
N2	1.05	0.99-1.12	0.11	1.08	1.01-1.15	0.03
N3	1.05	1.00-1.12	0.07	1.07	1.01-1.14	0.03
Unknown	1.23	1.14-1.33	<0.001	1.21	1.12-1.31	<0.001
Grade						
I	1.00	Reference		1.00	Reference	
II	1.21	1.13-1.31	<0.001	1.26	1.16-1.37	<0.001
III	1.64	1.52-1.78	<0.001	1.76	1.62-1.91	<0.001
IV	1.57	1.23-2.00	<0.001	1.67	1.29-2.15	<0.001
Unknown	1.42	1.31-1.54	<0.001	1.50	1.38-1.64	<0.001
Histology						
IDC 8500/3	1.00	Reference		1.00	Reference	
ILC 8520/3	1.10	1.04-1.17	0.001	1.13	1.07-1.20	<0.001
Mixed IDC/ILC 8522/3	1.02	0.94-1.10	0.68	1.04	0.95-1.13	0.44
Other types	1.07	1.02-1.13	0.01	1.06	1.00-1.12	0.04
Hormone receptor						
HR+/HER2-	1.00	Reference		1.00	Reference	
HR+/HER2+	0.89	0.84-0.93	<0.001	0.88	0.83-0.93	<0.001
HR-/HER2+	1.18	1.10-1.26	<0.001	1.17	1.09-1.25	<0.001
HR-/HER2-	2.63	2.51-2.77	<0.001	2.69	2.56-2.84	<0.001
Surgery						
Yes	1.00	Reference		1.00	Reference	
No	1.85	1.78-1.93	<0.001	1.91	1.81-2.00	<0.001
Unknown	1.63	1.32-2.02	<0.001	1.72	1.38-2.14	<0.001
Radiation						
Yes	1.00	Reference		1.00	Reference	
No/Unknown	1.00	0.96-1.03	0.91	0.97	0.93-1.01	0.09
Chemotherapy						
Yes	1.00	Reference		1.00	Reference	
No/Unknown	1.57	1.51-1.63	<0.001	1.54	1.48-1.61	<0.001

IDC, invasive ductal carcinoma; ILC, invasive lobular carcinoma.

## Discussion

The incidence of breast cancer is highly associated with increasing age (18). Emerging data also suggest that age is a key factor associated with cancer metastasis (12). Nevertheless, the patterns of age-related *de novo* metastatic breast cancer have not been established. In the present study, we systematically determined breast cancer metastasis at the time of diagnosis focusing on patient age based on an unbiased population cohort. We found that there are three peak incidences of *de novo* metastatic breast cancer including young (less than 40 years old), perimenopausal period (approximately 55 years old), and the oldest old ≥ 80 years old). Potential reasons for this finding may be due to a higher proportion of aggressive molecular subtypes (TNBC [18.35%] and HER-2 positive subtypes [24.78%]; **Figure 3**) in young patients, dramatical changes in hormone level and homeostasis during the perimenopausal period, and a decrease in immune surveillance among the oldest old (19, 20). Moreover, we also found that young Black patients were more frequently diagnosed with stage IV breast cancer, a finding which was consistent with the concept that Black patients have poor survival outcomes (21).

The distribution of metastatic sites was distinct among the four age groups. In this study, we found that patients older than 60 years of age had a much higher percentage of lung metastases, a phenomenon that may be due to chronic inflammation of the lung, such as chronic obstructive pulmonary disease, which is usually present in old people (22). It has been shown that potential mechanisms of inflammatory cell neutrophils specifically support metastatic initiation and promote dormant cancer cell awakening in the lungs using mouse models (23, 24). In addition, we observed that young patients had a higher prevalence of liver and bone metastases. A previous study reported that the HER2-positive subtype has a high rate of metastasis to the liver and bone, but the basal-like subtype has a significantly lower rate of liver and bone metastases (25). Considering the high percentages of HER-2 positive subtypes in the young cohort, further studies focusing on potential mechanisms of liver and bone metastases in HER-2 positive breast cancer may help in developing novel strategies for metastasis prevention. Brain metastases are associated with the worst prognosis in patients with breast cancer, with median survival ranging from 2 to 25.3 months despite treatment (26). In the present study, we observed that middle- and older-aged patients had a higher frequency of brain metastases. This finding is inconsistent with previous studies that reported that younger-aged patients are more likely to develop breast cancer brain metastases after diagnosis and treatment (27, 28). Moreover, multiple risk factors for brain metastases have also been identified, such as molecular subtype, histological grade, and germline BRCA1/2 mutations (28–30). Collectively, these data indicated that there was a close correlation between breast cancer metastasis and patient age and that different ages exhibited distinct metastatic patterns.

Young patients with early breast cancer typically have more aggressive subtypes, which are more likely to develop both locoregional and distant recurrences and are usually associated with poorer survival outcomes, whereas older women more commonly have less aggressive tumors with a better prognosis (31). In contrast, our multivariable analysis revealed that older patients with *de novo* metastatic breast cancer were more likely to have a poorer OS and BCSS than young women because a lower percentage of older patients received therapy, including surgery, radiotherapy, and chemotherapy, than young patients, which was consistent with a previous study in which elderly patients tended to receive less chemotherapy or fewer treatment regimens than younger patients (32). It is clear that surgery, radiotherapy, and chemotherapy significantly increased the OS and BSCC in the four age groups compared with patients who did not receive surgery, radiotherapy, or chemotherapy for *de novo* metastatic breast cancer in the SEER cohort (33, 34). Among patients who received surgery, radiotherapy, or chemotherapy, the young patients had the best OS and BCSS, followed by the middle-aged, older, and oldest old patients. This finding indicated that older patients with *de novo* metastatic breast cancer did not acquire more benefits than younger patients from these treatments. Moreover, a SEER database-based retrospective study reported that older patients (i.e., >70 years) had a higher risk of death from heart disease (32). Therefore, additional strategies involving non-cancer causes of death might enhance the OS and BCSS in patients with *de novo* metastatic breast cancer, especially in older patients.

A recent study reported that lymph node involvement and T stage were independent risk factors for mortality in the population with *de novo* metastatic breast cancer (4). In the present study, we also showed that the locoregional tumor burden (tumor size and lymph node status) decreased with aging, which implied that older patients might develop distant metastases earlier than younger patients. Different distant metastases were associated with distinct survival outcomes in breast cancer, of which brain metastasis is the leading cause of death (35). Our subgroup analyses also detected the impact of age on different metastatic organs. We found that older patients had poorer OS and BCSS compared to younger patients with bone, liver, lung, and brain metastases, except that no OS and BCSS differences were observed between young- and middle-aged patients with lung and brain metastases. Moreover, older patients were associated with a poorer OS and BCSS than younger patients with different molecular subtypes of breast cancer, including HR+/HER2-, HR-/HER2+, and HR-/HER2-. Although there were no significant OS and BCSS differences between young- and middle-aged patients with TNBC, the oldest old patients had a poorer OS and BCSS compared to the other patients. The results of the subgroup analysis showed the robustness of our findings, which suggested that older patients were associated with a poorer OS and BCSS compared to younger patients with *de novo* metastatic breast cancer.

There were some limitations to our study. First, potential selection bias could not be avoided due to the properties of a retrospective study and the exclusion of patients because of the lack of essential information. Second, the SEER database did not provide historical information on accompanying diseases, such as cardiovascular and chronic obstructive pulmonary diseases; therefore, we could not adjust for these factors when performing the multivariable analysis. Third, because the detailed systemic treatment information, such as chemotherapy, endocrine therapy, and targeted therapy, was not available in the SEER database, we could not investigate the benefits of systemic treatment in different age groups.

## Conclusions

In conclusion, age was a critical factor that was closely correlated with *de novo* metastatic breast cancer. Different ages had distinct distant metastasis patterns at the time of diagnosis. Older patients with *de novo* metastatic breast cancer had poorer OS and BCSS compared to young women. Future basic research and clinical studies may help to disclose novel mechanisms of how age influences distant metastasis and develop personalized treatment strategies for patients with *de novo* metastatic breast cancer in different ages.

## Data availability statement

The original contributions presented in the study are included in the article/**Supplementary Material**. Further inquiries can be directed to the corresponding authors.

## Ethics statement

The requirement of ethical approval was waived by the Institutional Ethics Committees of the Women and Children’s Hospital of Chongqing Medical University for the studies involving humans because no participants were enrolled in this study. The studies were conducted in accordance with the local legislation and institutional requirements. The ethics committee/institutional review board also waived the requirement of written informed consent for participation from the participants or the participants’ legal guardians/next of kin because no participants were enrolled in this study.

## Author contributions

QX: Writing - Original Draft; LS: Writing - Review & Editing; WZ, JJ, and ZD: Data Curation; DL, JZ, PL, and TZ: Visualization; HG: Writing - Review &Editing, Methodology, Supervision. All authors contributed to the article and approved the submitted version.

## Supplementary material

Supplementary Figure 1 Overall survival of *de novo* metastatic breast cancer stratified by subtypes. HR+/HER2- **(A)**, HR+/HER2+ **(B)**, HR-/HER2+ **(C)**, and HR-/HER2- **(D)**.

Supplementary Figure 2 Breast cancer-specific survival of *de novo* metastatic breast cancer stratified by subtypes. HR+/HER2- **(A)**, HR+/HER2+ **(B)**, HR-/HER2+ **(C)**, and HR-/HER2- **(D)**.

Supplementary Figure 3 Overall survival of *de novo* metastatic breast cancer stratified by distribution of metastatic sites. Bone **(A)**, liver **(B)**, lung **(C)**, and brain **(D)**.

Supplementary Figure 4 Breast cancer-specific survival of *de novo* metastatic breast cancer stratified by distribution of metastatic sites. Bone **(A)**, liver **(B)**, lung **(C)**, and brain **(D)**.

Supplementary Figure 5 Overall survival and breast cancer-specific survival of *de novo* metastatic breast cancer patients who received chemotherapy. **(A)** Overall survival curves. **(B)** Breast cancer-specific survival curves.

Supplementary Figure 6 Overall survival and breast cancer-specific survival of *de novo* metastatic breast cancer patients who underwent surgery. **(A)** Overall survival curves. **(B)** Breast cancer-specific survival curves.
