# Supplementary figures and images for: Patterns of de novo metastasis and survival outcomes by age in breast cancer patients: a SEER population-based study
Source: Front Endocrinol (Lausanne). 2023 Nov 6;14:1184895. doi: 10.3389/fendo.2023.1184895 (PMC10657995; doi:10.3389/fendo.2023.1184895)

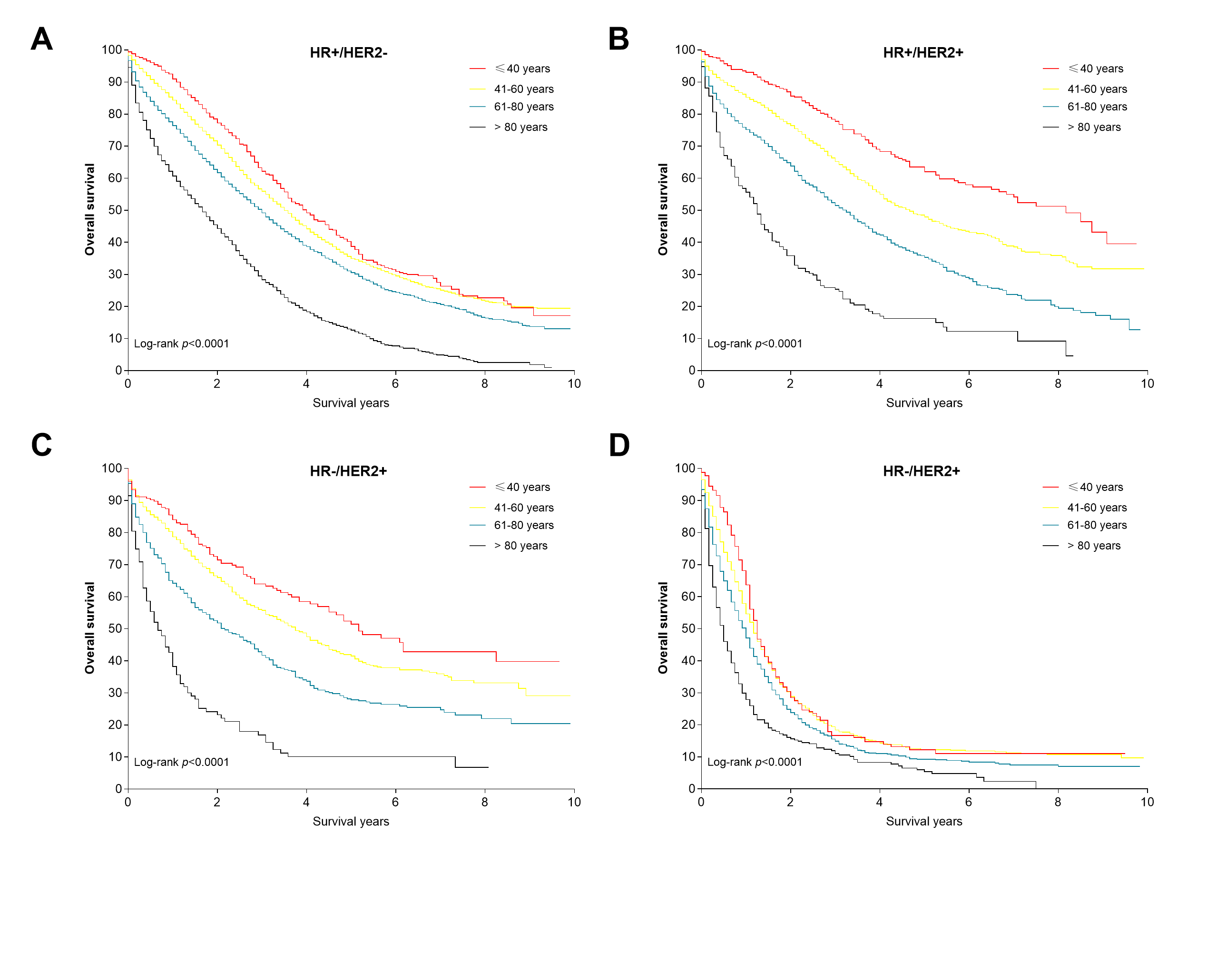

Supplement: Supplementary Figure 1 — Overall survival of de novo metastatic breast cancer stratified by subtypes. HR+/HER2- (A), HR+/HER2+ (B), HR-/HER2+ (C), and HR-/HER2- (D). [file Image_1.tif]

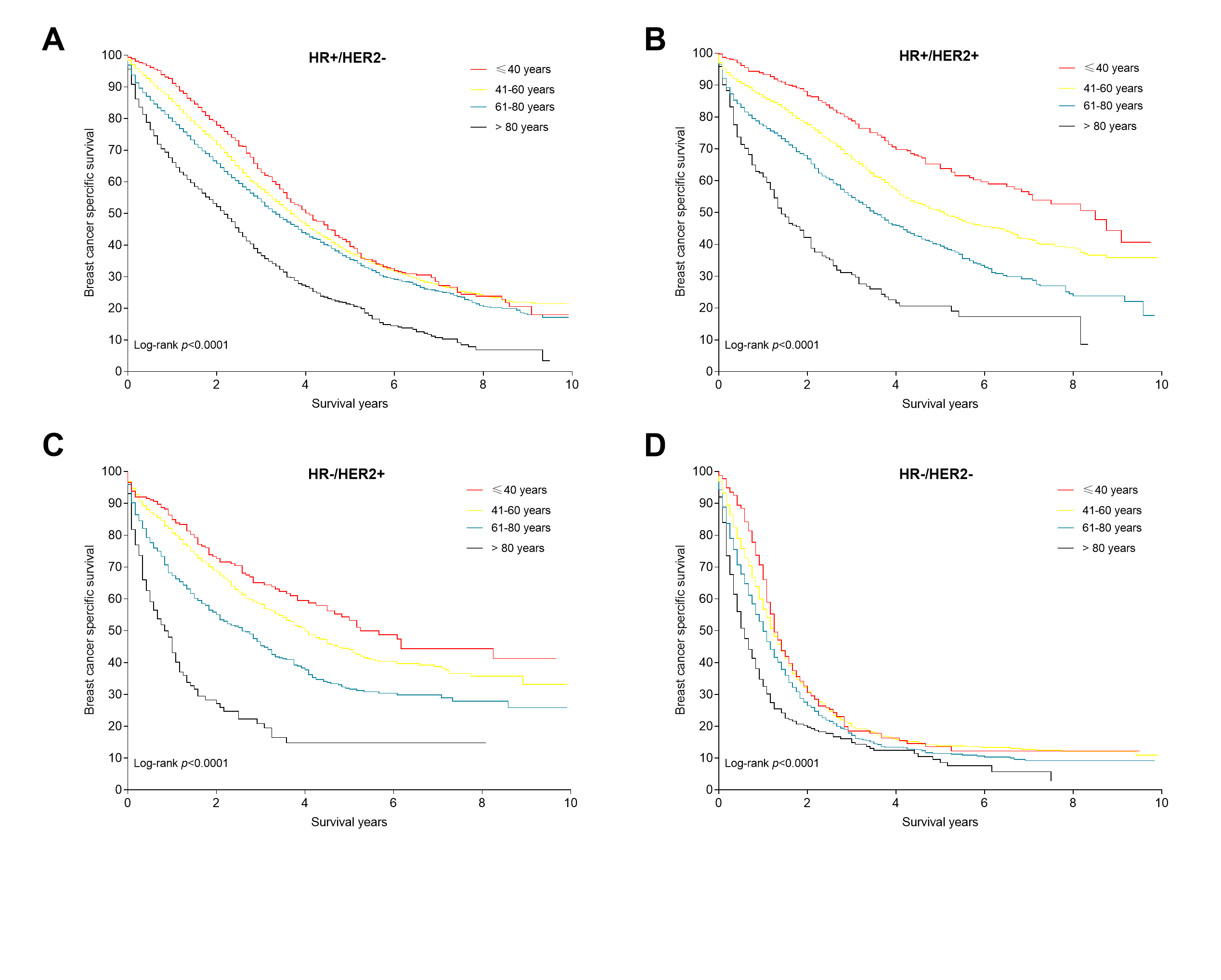

Supplement: Supplementary Figure 2 — Breast cancer-specific survival of de novo metastatic breast cancer stratified by subtypes. HR+/HER2- (A), HR+/HER2+ (B), HR-/HER2+ (C), and HR-/HER2- (D). [file Image_2.tif]

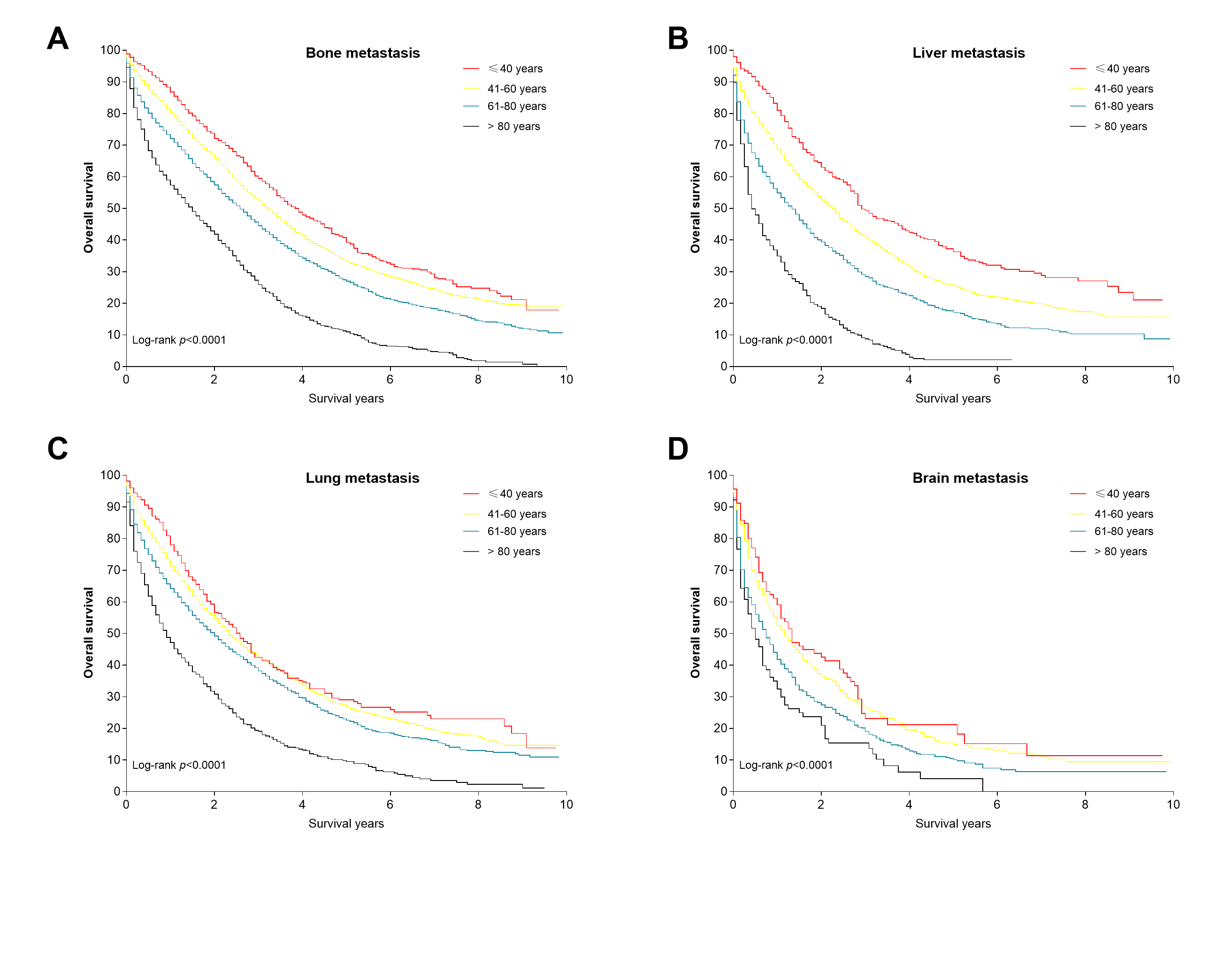

Supplement: Supplementary Figure 3 — Overall survival of de novo metastatic breast cancer stratified by distribution of metastatic sites. Bone (A), liver (B), lung (C), and brain (D). [file Image_3.tif]

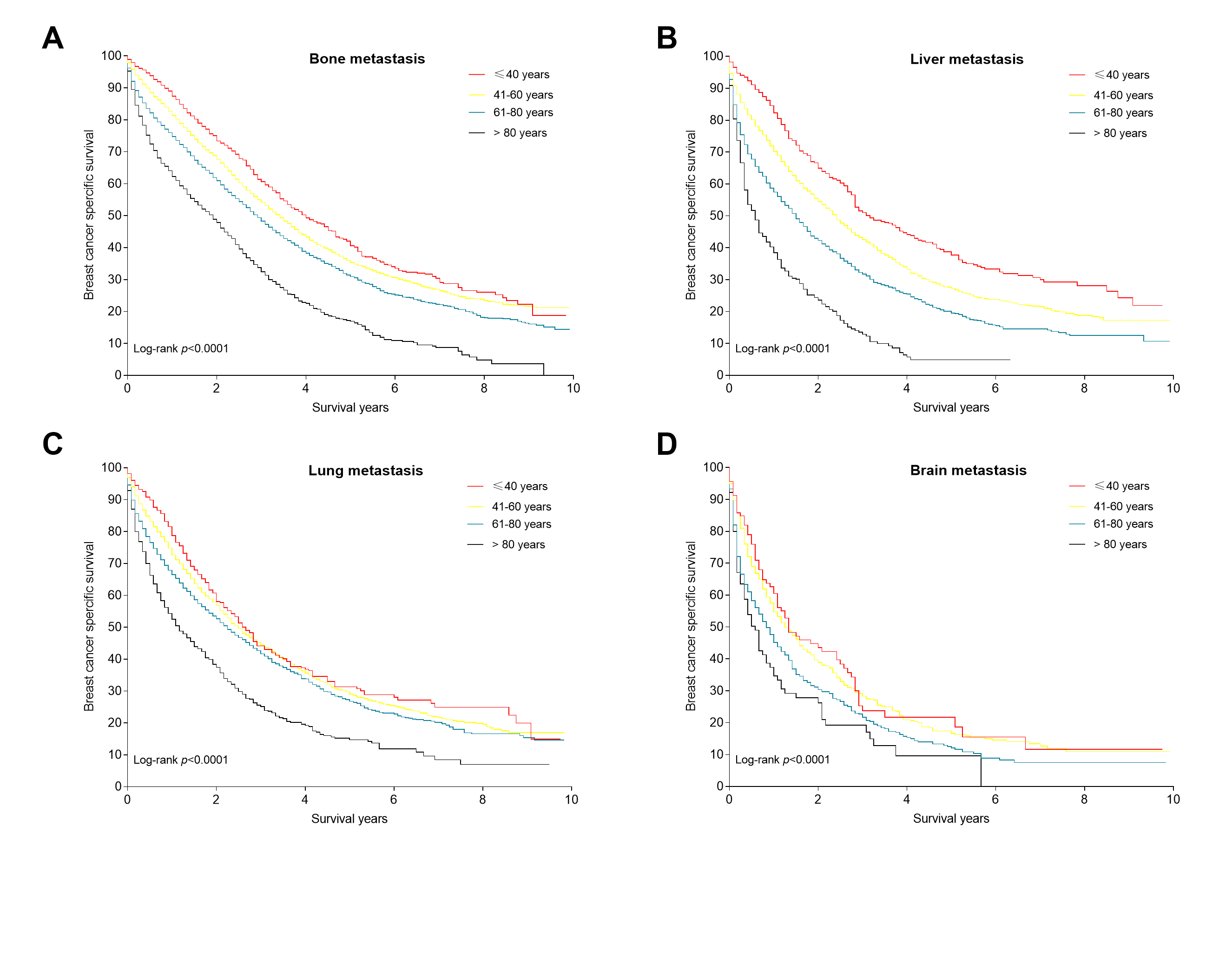

Supplement: Supplementary Figure 4 — Breast cancer-specific survival of de novo metastatic breast cancer stratified by distribution of metastatic sites. Bone (A), liver (B), lung (C), and brain (D). [file Image_4.tif]

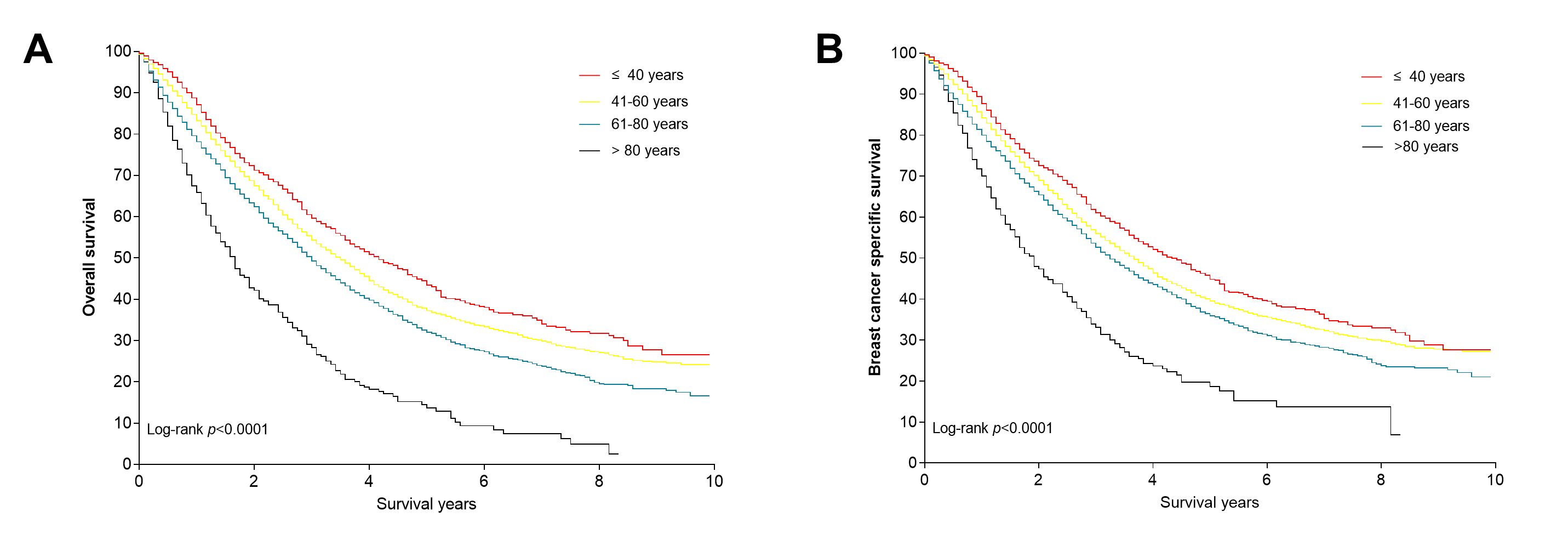

Supplement: Supplementary Figure 5 — Overall survival and breast cancer-specific survival of de novo metastatic breast cancer patients who received chemotherapy. (A) Overall survival curves. (B) Breast cancer-specific survival curves. [file Image_5.tif]

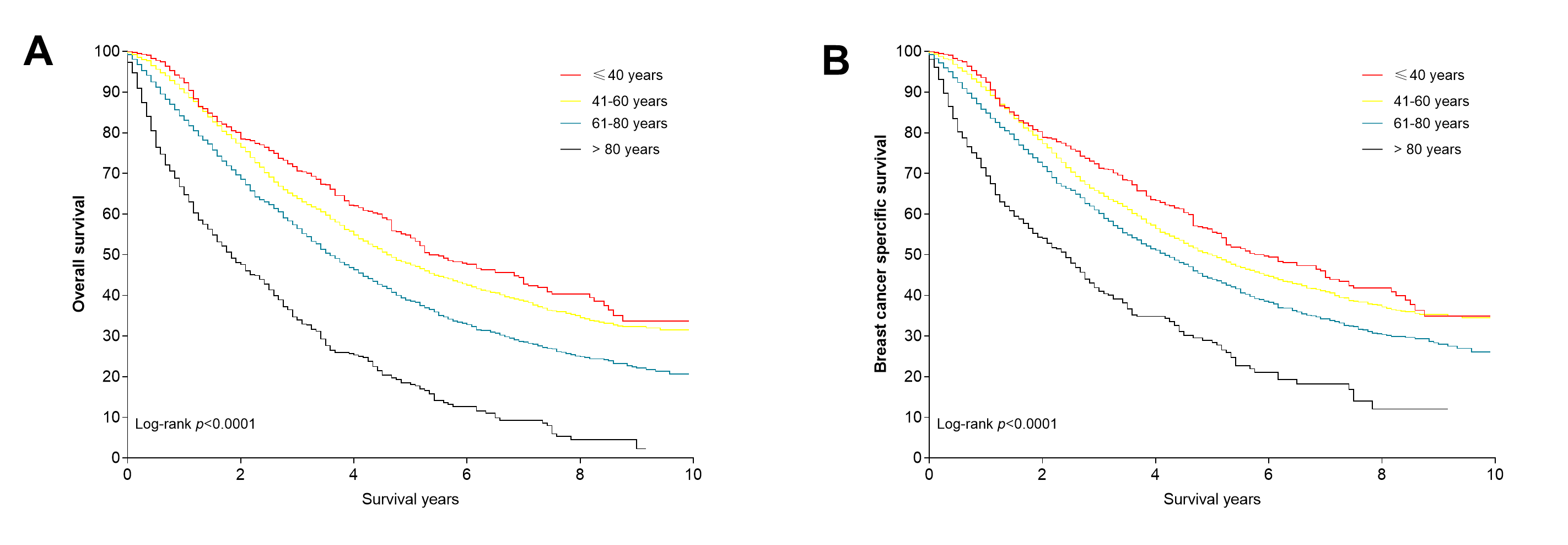

Supplement: Supplementary Figure 6 — Overall survival and breast cancer-specific survival of de novo metastatic breast cancer patients who underwent surgery. (A) Overall survival curves. (B) Breast cancer-specific survival curves. [file Image_6.tif]
